# Dietary intake and sources of sodium and potassium, and salt-related knowledge, attitudes, and behaviours among rural adults in China: a cross-sectional study

**DOI:** 10.3389/fnut.2026.1861183

**Published:** 2026-06-11

**Authors:** Beike Wu, Jianwei Xu, Zhifang Li, Anqi Ge, Min Liu, Yuze Xin, Shuangjie Peng, Qing Li, Tengyi Wang, Xuejun Yin, Xinyan Liu, Jing Zhang, Xinyi Zhang, Kathy Trieu, Bruce Neal, Katrina Kissock, Maoyi Tian

**Affiliations:** 1 School of Public Health, Harbin Medical University, Harbin, China; 2 National Centre for Chronic and Noncommunicable Disease Control and Prevention, Chinese Centre for Disease Control and Prevention, Beijing, China; 3 School of Preventive Medicine, Changzhi Medical College, Changzhi, China; 4 School of Public Health, Jiangxi Medical College, Nanchang University, Nanchang, Jiangxi, China; 5 Jiangxi Provincial Key Laboratory of Disease Prevention and Public Health, Nanchang University, Nanchang, China; 6 The George Institute for Global Health, Faculty of Medicine and Health, University of New South Wales, Sydney, NSW, Australia; 7 School of Public Health, Imperial College London, London, United Kingdom; 8 Department of General Practice, The Second Affiliated Hospital of Harbin Medical University, Harbin, China; 9 National Health Commission Key Laboratory in Etiology and Epidemiology, Harbin Medical University, Harbin, China; 10 Center for Endemic Disease Control, Chinese Center for Disease Control and Prevention, Harbin Medical University, Harbin, China

**Keywords:** 24-h urine, dietary potassium, dietary sodium, non-communicable diseases (NCDs), rural adults, salt reduction strategies, salt-related KAB

## Abstract

We assessed dietary intake and sources of sodium and potassium, and salt-related knowledge, attitudes, and behaviours (KAB) among rural adults in four Chinese provinces. This cross-sectional study was conducted in four provinces in China, with two counties per province and eight villages per county purposively selected. Participants (18–69 years) were enrolled using stratified random sampling by sex and age in each village. Sodium and potassium intake were estimated using eligible 24-h urine samples, with a 24-h dietary recall collected from a 25% subsample to assess dietary sources. Salt-related KAB was assessed using an interviewer-administered questionnaire. Data were summarized using sampling weights as means (SE) for continuous variables and weighted proportions for categorical variables. A total of 2,669 participants were recruited between August 2021 and May 2023. Mean sodium and potassium intake was 5.8 g/day (SE 0.1) and 2.39 g/day (SE 0.04), respectively, equivalent to 14.7 g/day of salt (SE 0.2). Overall, 77.7% of participants were aware of the health risks of excessive salt or salty sauce intake, 67.3% recognized the importance of reducing salt in cooking, and 50.1% reported trying to reduce salt intake. We find that 72.1% of sodium intake came from salt added during home cooking, while cereals and tubers were the main sources of potassium. Rural Chinese adults consume excessive sodium and insufficient potassium relative to World Health Organization recommendations, with generally poor attitudes and adoption of salt-reduction behaviours. These findings highlight the need for interventions that strengthen salt-reduction education, provide practical tools, and improve supportive food environments.

## 1 Introduction

Excessive sodium intake is a well-established risk factor for non-communicable diseases (NCDs) (1). The World Health Organization (WHO) recommends that adults consume no more than 2 g/day of sodium (5 g/day salt) (2). Recent evidence based on 24-h urine collections indicates that adult sodium intake typically ranges from 3.3 to 4.9 g/day globally (3), with China reporting mean levels of approximately 4.3 g/day (4). In China, overconsumption of sodium leads to an estimated annual 1.7 million disability-adjusted life years (DALYs), and represents a major opportunity for chronic disease prevention (5).

In response to the public health challenge of excess sodium intake, the Chinese government launched a series of national policies, including the National Nutrition Plan (2017–2030) (6) and the Healthy China Action Plan (2019–2030) (7), that aim to reduce average daily sodium intake by 20% by 2030. Achieving this target will require tailored interventions informed by current data about sodium intake levels, dietary sources of sodium, and salt-related knowledge, attitudes, and behaviours (KAB) (8). However, existing research in China predominantly relies on indirect methods (e.g., dietary recalls, food records, or spot urine samples) to estimate sodium intake rather than 24-h urine collections, the gold-standard method for sodium assessment (9, 10). In addition, few have simultaneously described dietary sodium intake, sources and salt related KAB limiting comprehensive understanding (4, 11).

Rural China is defined here as administrative villages classified as rural by the National Bureau of Statistics of China. This classification typically encompasses areas characterized by lower population density, a predominance of agricultural livelihoods, and limited access to urban infrastructure and services (12). As of 2022, rural residents. Accounted for 36.1% of the national population (13). Compared with urban populations, rural residents often experience poorer dietary behaviours largely attributable to lower education levels and household income, reduced access to healthcare and nutrition education, and greater reliance on traditional home cooking practices with discretionary salt use (13, 14). Therefore, high-quality studies focusing on rural China are urgently needed to inform targeted strategies.

Insufficient potassium intake is another cause of high blood pressure and the effects of sodium and potassium are physiologically interlinked, with higher potassium intake facilitating sodium excretion through sodium homeostasis (15). Accordingly, combined sodium reduction and potassium increase provide greater blood pressure-lowering benefits than either intervention alone (16). Chinese adults consume only 1.5 g/day of potassium (17), less than half the WHO recommendation of 3.5 g/day (18). Assessing dietary potassium consumption alongside dietary sodium provides an opportunity to optimisethe impact of dietary intervention strategies on blood pressure.

Evaluation of Population-based Sodium Reduction Strategies in China is a study that uses repeated independent cross-sectional surveys to investigate the effectiveness of government-led sodium reduction programs implemented in China. The study employs a combination of quantitative and qualitative approaches, consisting of population surveys, food retail surveys, and stakeholder interviews at baseline and follow-up (19). Here we use baseline population survey data to estimate the dietary intake and dietary sources of sodium and potassium, as well as the salt-related KAB among rural adults in four Chinese provinces.

## 2 Materials and methods

This study was a cross-sectional analysis (19). Approval to conduct the study was received from the University of New South Wales Human Research Ethics Committee (HC200413), the Ethics Committee of the National Centre for Non-Communicable Disease Prevention and Control, Chinese Centre for Disease Prevention and Control (202021–01) and Changzhi Medical College Ethics Committee (RT2020023). All participants provided written informed consent.

### 2.1 Study setting

**Figure 1:**
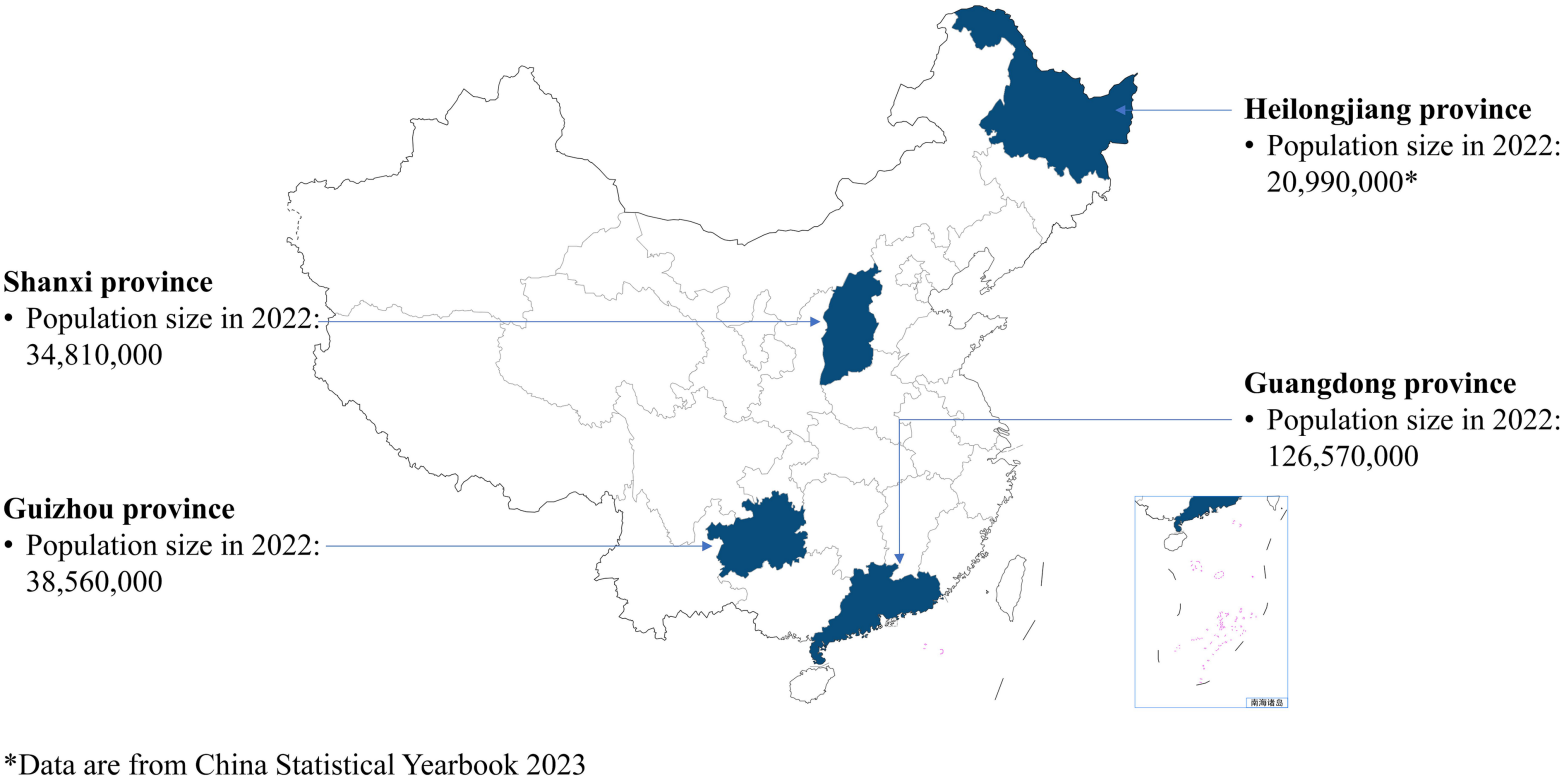
Geographical locations of four study sites.

This study was conducted in eight counties from four Chinese provinces (two counties per province): Shanxi Province, Heilongjiang Province, Guizhou Province, and Guangdong Province (Figure 1). These provinces were selected to represent different regions of China, including North China, Northeast China, South China, and Southwest China, and to reflect varying socioeconomic and dietary characteristics. In each county, eight villages were purposively selected, resulting in a total of 64 villages participating in the study. Villages were selected primarily based on proximity to the research team and their willingness to participate, while also ensuring that villages from different local areas within each county were included to provide diversity. Villages in China serve as the fundamental organizational unit for the rural population. Each village has defined boundaries, a designated head, and a cluster of residents, but population size and land area vary.

### 2.2 Study participants

Participants aged 18 to 69 years who were permanent residents of the selected villages were eligible for recruitment. Individuals above 69 years were not included in the study primarily for practical and safety considerations, as completing 24-h urine collections in older adults may present additional challenges, and the recruitment and consent procedures for this age group require additional precautions. Participants who were pregnant, highly dependent on medical care, or unable to give consent were excluded from the study. A recruitment list was generated through random selection of age-eligible village members using stratified random sampling by sex (female, male) and age group (18–44 and 45–69 years) in each village. In Heilongjiang, Guangdong, and Guizhou provinces, approximately 30 participants were planned to be recruited from each village, with broadly comparable recruitment targets across sex and age groups. Shanxi Province, as the first implementation site, expanded the sample size during the project initiation phase and adopted a differentiated age-stratified allocation. Specifically, the target sample size for each sex stratum was 14 in the 18–44 years age group and 8 in the 45–69 years age group. This allocation was designed to achieve balanced representation across age and sex strata while accounting for the village population structures, anticipated participation rates, and logistic. Village doctors, who served as primary health care providers at the village level, were then asked to invite participants according to the recruitment list. If a selected participant declined or was unavailable on the survey day, the next eligible individual on the list was invited. In addition, a 24-h dietary recall was conducted among a randomly selected 25% subsample of the recruited participants in each village, using the same stratified random sampling approach as applied in the initial recruitment.

### 2.3 Data collection

Data were collected from all participants through interviewer-administered questionnaires, anthropometric measurements, spot urine samples, and 24-h urine samples. Interviewer-assisted 24-h dietary recall questionnaires were done with the subsample.

The interviewer-administered questionnaire was adapted from the INTERSALT study (20) a standardized international framework widely used in population-based salt intake research, and was further modified according to the dietary habits, language expression, and salt-related practices commonly observed in rural Chinese populations to improve cultural appropriateness and field applicability. The questionnaire consisted of three sections including demographic characteristics (e.g., sex, age, education level, smoking history, disease and medicine history), quality of life assessment using EuroQol-5 Dimension (EQ-5D), and salt-related KAB. The EQ-5D questionnaire comprised a graded visual analog scale (VAS) assessed by participants themselves from 0 (the worst possible health status) to 100 (the best possible health status). The EuroQol 5-Dimension 5-Level (EQ-5D-5L) was used to generate an EQ-5D index utility score anchored at 0 for death and 1 for perfect health (21).

Anthropometric measures including height, weight, waist circumference, and blood pressure (BP) were collected. Height and weight were measured without shoes and in light clothing using a calibrated stadiometer and digital scale (22), and waist circumference was measured at the midpoint between the lowest rib and the iliac crest (23). Body Mass Index (BMI) was calculated as weight (kg) divided by height (m)^2^ and categorized according to the criteria of the Chinese Working Group on Obesity: underweight (BMI < 18.5 kg/m^2^), normal weight (18.5–24.0 kg/m^2^), overweight (24.0–28.0 kg/m^2^), and obese (≥28.0 kg/m^2^) (24). Blood pressure (BP) was measured three times using an Omron electronic sphygmomanometer with 5 min of rest between measurements, and the mean of the three readings was used to define systolic blood pressure (SBP) and diastolic blood pressure (DBP) (25). Participants with a SBP of ≥130 mmHg and/or a DBP of ≥80 mmHg were classified as being at increased risk of hypertension (26).

We then collected spot urine and 24-h urine samples from all participants. For spot urine collection, participants were provided with a disposable cup and asked to collect a midstream sample from which two 2-mL aliquots were prepared for testing. The time of spot urine collection was recorded as the starting point for the 24-h urine collection. For 24-h urine collections, participants collected all urine excreted over the subsequent 24 h in 1000-mL containers provided by the researchers and returned the following day. All samples were then combined in a single container, and the total volume and self-reported missed volume were recorded. Two 2-mL aliquots of the pooled 24-h urine sample were then prepared for laboratory analysis. Urinary sodium (Na^+^) and potassium (K^+^) concentrations were determined using the ion-selective electrode method, and urinary creatinine (Cr) was measured using the sarcosine oxidase method on a HITACHI 7600 automated biochemistry analyzer (Hitachi Co., Tokyo, Japan).

Finally, we conducted a 24-h dietary recall concurrently with urine collection for a 25% subsample from each village to balance logistical feasibility, interviewer workload, and participant burden. The dietary recall was intended to correspond to the same 24-h period as the urine collection. Participants were provided with a booklet to record all foods and beverages consumed at each meal time (breakfast, lunch, dinner), cooking methods, and details of both main foods and condiments used in mixed dishes, together with the quantity of each item. The completed booklets were collected the following day, and trained researchers then conducted face-to-face interviews, using the booklet to complete the 24-h dietary recall data.

### 2.4 Sample size calculation

A sample size of at least 1,920 participants from 64 villages was determined to provide 90% power (*α* = 0.05) to detect a reduction of 0.4 g/day or greater in mean sodium intake between this baseline survey and the planned follow-up survey. The power assumption assumes a baseline mean intake of 4.4 g/day with a standard deviation of 3.8 g/day (27). Although the present manuscript reports only baseline data, the planned sample size provides adequate precision for estimating mean sodium and potassium intake, characterizing food source contributions, and describing salt-related KAB patterns at baseline. We present this calculation for completeness and transpar ency regarding the parent study design.

### 2.5 Statistical analysis

Continuous variables are presented as weighted means with standard errors (SEs), and categorical variables are summarized as weighted percentages. Salt-related KAB variables were reported as categorical variables. Overall differences across the four groups were assessed using survey-weighted linear regression models for continuous variables and the Rao–Scott chi-square test for categorical variables. All continuous and categorical variables were compared across provinces, except for food source contributions as these data are compositional in nature (the proportions sum to 100%) and the components are not independent (28, 29). Instead, descriptive statistics were reported to illustrate provincial variation in dietary sodium and potassium sources, providing a clear overview of regional patterns without implying inferential testing. When a significant overall group effect was observed, pairwise comparisons for continuous variables were performed using Tukey–Kramer-adjusted contrasts based on survey-weighted linear regression models. For categorical outcomes requiring detailed between-group comparisons, survey-weighted logistic regression models with pairwise contrasts were additionally applied. A *p*-value < 0.05 was considered statistically significant.

For the spot urine analyses the sodium and potassium concentrations were expressed in mmol/L. For the 24-h urine analyses the sodium and potassium excretion was calculated as follows: (1) sodium (g/day) = (23 (mg/mmol) × concentration (mmol/L) × 24-h urine volume (L/day)) / 1,000; and (2) potassium (g/day) = (39.1 (mg/mmol) × concentration (mmol/L) × 24-h urine volume (L/day)) / 1,000 (30). Salt (g/day) was calculated as sodium (g/day) × 2.54. The 24-h urine samples were excluded if any of the following criteria were met, consistent with prior studies (30): (1) self-reported missed urine volume exceeded 10% of the total volume; (2) total 24-h urine volume was < 500 mL or > 6,000 mL; (3) 24-h urine creatinine excretion was < 4 mmol or > 25 mmol in women, or < 6 mmol or > 30 mmol in men; or (4) urine collection duration was < 22 or > 26 h (31, 32). Approximately 7% of sodium and 23% of potassium are excreted via non-urinary routes (3, 18, 33). Accordingly, estimated dietary sodium and potassium intakes were calculated from valid 24-h urine samples using correction factors commonly applied in population-based sodium and potassium intake assessment studies (urinary sodium/0.93; urinary potassium/0.77). We report both dietary sodium, potassium and salt intake (adjusted values) and the urine sodium, potassium and Salt equivalent based on urine sodium (original urinary excretion values).

For the analysis of 24-h dietary recalls, the dietary intake of sodium and potassium was calculated based on the Chinese Food Composition Table (34, 35). The percentage contribution of each food category to daily sodium and potassium intake was calculated by summing the sodium and potassium contents for each food category separately and dividing by the total sodium and potassium contents of the diet, respectively. Percent contribution to sodium and potassium intake were calculated for the following 12 food groups: (1) salt, (2) soy sauce, (3) monosodium glutamate (MSG) and chicken essence, (4) animal meat (meat, poultry and game, fish and shrimp), (5) vegetables and fruits, (6) pickled vegetables, (7) processed foods (instant noodles, deep-fried dough sticks and cakes, steamed stuffed buns, dumplings, baked pancakes, breads, and others), (8) eggs, (9) legumes, (10) cereals and tubers, (11) other condiments (vinegar and sauces), and (12) others (wine, dairy, beverage, seaweed, edible oil, nuts) (36–38).

To enhance the representativeness of provincial estimates, Post-stratification weights were constructed using the age–sex distribution of the village population in each province from the 2020 China Population Census (39), the most recent authoritative population data available. Participants were stratified by sex and age group (18–44 and 45–69 years), and sampling weights were calculated as the ratio of the population proportion to the corresponding sample proportion within each stratum. Weights were derived separately for the questionnaire, spot urine, 24-h urine, and 24-h dietary recall datasets to account for differential missingness across study components (Supplementary Tables S1–S4). Because the official population statistics were reported in 5-year age bands, proportional redistribution within age groups was applied under the assumption of a uniform age distribution when the target age categories did not align exactly with the published bands. Specifically, the population aged 18–19 years was estimated as two-fifths of the reported population aged 15–19 years and combined with adjacent age groups to derive the 18–44 years population.

To assess the potential selection bias arising from incomplete or ineligible 24-h urine collections, supplementary comparisons between participants with eligible and ineligible 24-h urine samples were conducted using survey-weighted statistical methods consistent with those described above. Similar comparisons were also performed between participants included in the 24-h dietary recall subsample and those not included in the dietary recall assessment to evaluate the representativeness of the dietary subsample.

All statistical analyses were conducted using SAS software (version 9.3; SAS Institute Inc., Cary, NC, USA) and Python software (version 3.9.13; Python Software Foundation, Wilmington, DE, USA).

## 3 Results

### 3.1 Participant enrollment and data collection

**Figure 2:**
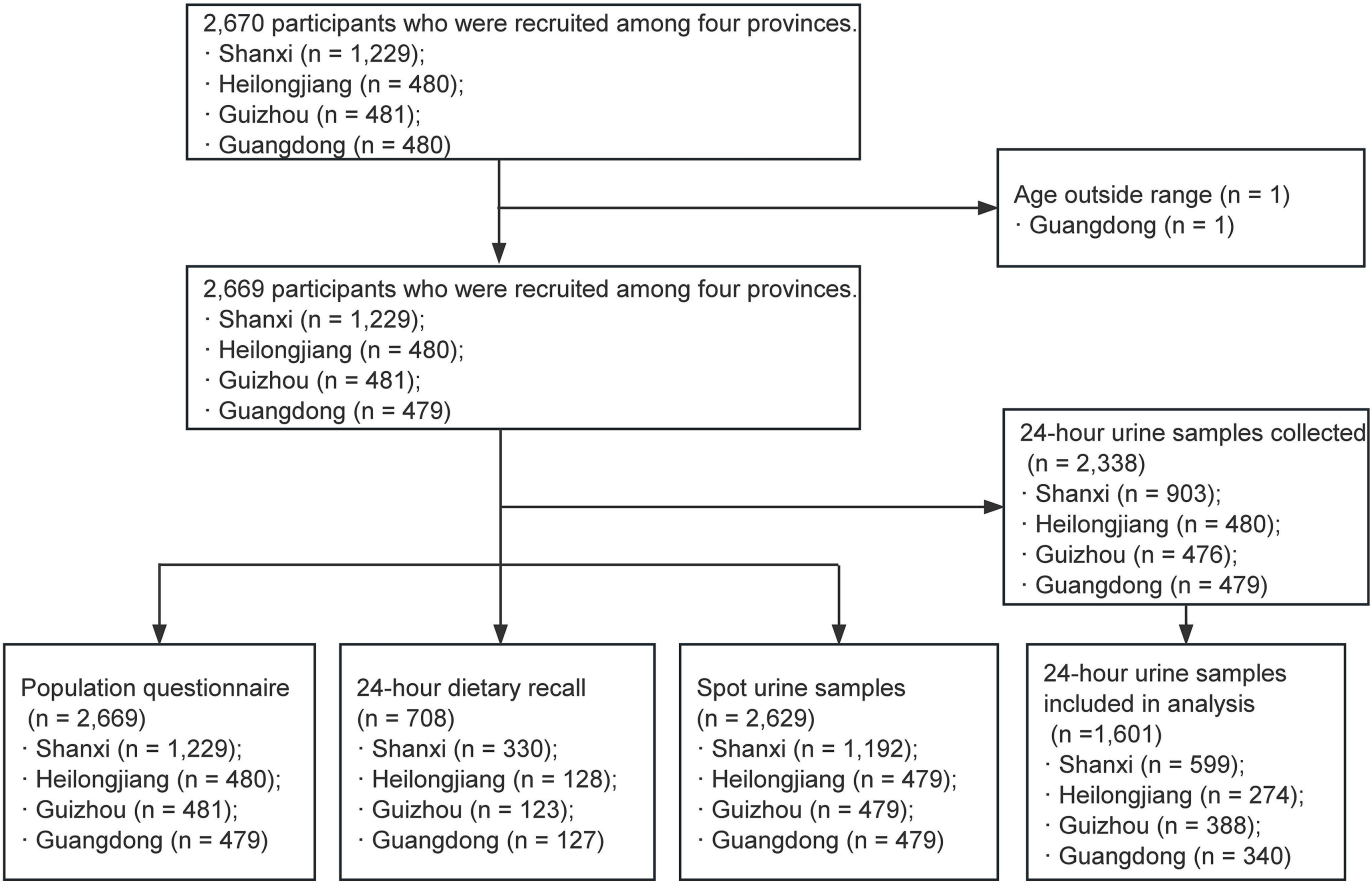
Flowchart of the study.

Data were collected between August 2021 and May 2023. A total of 2,669 participants were recruited: 1,229 (46.1%) in Shanxi, 480 (18.0%) in Heilongjiang, 481 (18.0%) in Guizhou, and 479 (17.9%) in Guangdong. Overall, 98.5% completed a spot urine collection and 60.0% provided an eligible 24-h urine sample. Additionally, 26.9% completed the 24-h dietary recall (Figure 2).

### 3.2 Characteristics of study participants

The mean age of all participants was 47.7 years (SE 0.3), 48.7% were female, and the mean BMI was 24.7 kg/m^2^ (SE 0.1) (Table 1). Most participants (73.7%) had an educational level of primary school or lower. Overall, 31.6% of participants were current smokers and 34.0% were former smokers. The mean SBP and DBP were 129.0 mmHg (SE 0.4) and 81.9 mmHg (SE 0.2), respectively. The mean EQ-5D utility score and EQ-VAS score were 0.994 (SE 0.001) and 82.6 (SE 0.2), respectively. Initial comparisons across the four provinces showed statistically significant differences in age, BMI, education level, smoking status, SBP, DBP, and health-related quality of life (all *p* < 0.05).

**Table 1:** Weighted demographic characteristics of rural adults in four Chinese provinces.

Characteristics	Total	Shanxi	Heilongjiang	Guizhou	Guangdong	*p*-value
Number (*n*)	2,669	1,229	480	481	479	
Sex, Female (%)	48.7	49.4	46.8	46.5	47.1	0.613
Age (years), mean (SE)	47.7 (0.3)	49.5 (0.4)^a^	47.5 (0.5)^b^	46.1 (0.6)^bc^	44.5 (0.6)^c^	<0.001
18–44 (%)	40.1	33.5ᵃ	35.8ᵃ	47.7ᵇ	53.8ᵇ	<0.001
45–69 (%)	59.9	66.5ᵃ	64.2ᵃ	52.3ᵇ	46.2ᵇ	
BMI (kg/m^2^), mean (SE)	24.7 (0.1)	24.9 (0.1)^a^	25.6 (0.2)^b^	24.2 (0.2)^c^	23.8 (0.2)^c^	<0.001
< 18.5 (%)	3.1	2.5ᵃ	1.4ᵃ	4.1ᵃ	5.4ᵇ	<0.001
18.5–24.0 (%)	41.7	39.4ᵃ	31.6ᵇ	46.3ᵃᶜ	53.4ᶜ	
24.0–28.0 (%)	39.2	42.4ᵃ	44.4ᵃ	34.8ᵇ	29.8ᵇ	
≥ 28.0 (%)	16.0	15.7ᵃ	22.5ᵇ	14.8ᵃ	11.5ᵃ	
Education (%)						
Primary school and lower	73.7	74.3ᵃ	85.1ᵇ	75.2ᵃ	59.1ᶜ	<0.001
Junior high school and above	26.3	25.7ᵃ	14.9ᵇ	24.8ᵃ	40.9ᶜ	
Smoking (%)						
Never smoked	65.7	67.1ᵃᵇ	69.3ᵇ	60.8ᵃ	63.5ᵃᵇ	0.023
Ever smoking^*^	34.0	32.5ᵃ	30.7ᵃ	39.0ᵇ	36.3ᵃᵇ	0.022
Current smoking^†^	31.6	29.6ᵃ	29.5ᵃ	35.2ᵇ	35.3ᵇ	0.035
Waist circumference (cm), mean (SE)	86.0 (0.2)	88.2 (0.3)ᵃ	87.0 (0.5)ᵃ	81.8 (0.5)ᵇ	83.9 (0.4)ᶜ	<0.001
SBP (mm Hg), mean (SE)^^^	129.0 (0.4)	127.8 (0.6)^a^	136.4 (1.0)^b^	126.3 (0.8)^a^	127.4 (0.8)^a^	<0.001
< 130 (%)	57.0	57.0ᵃ	43.5ᵇ	65.2ᵃ	62.6ᵃ	<0.001
≥130 (%)	43.0	43.0ᵃ	56.5ᵇ	34.8ᵃ	37.4ᵃ	
DBP (mm Hg), mean (SE)^^^	81.9 (0.2)	81.7 (0.3)^a^	84.9 (0.5)^b^	80.6 (0.6)^a^	80.8 (0.5)^a^	<0.001
< 80 (%)	44.7	44.4ᵃ	32.1ᵇ	50.6ᵃᶜ	52.1ᶜ	<0.001
≥ 80 (%)	55.3	55.6ᵃ	67.9ᵇ	49.4ᵃᶜ	47.9ᶜ	
Disease history (%)						
Hypertension	21.8	25.1ᵃ	23.2ᵃ	22.6ᵃ	10.7ᵇ	<0.001
Diabetes mellitus	5.9	5.3ᵃ	9.5ᵇ	6.3ᵃᵇ	3.0ᶜ	<0.001
Transient ischemic attack	2.7	2.4ᵃ	6.6ᵇ	1.5ᵃ	0.8ᵃ	<0.001
Ischemic heart disease	2.0	1.4ᵃ	5.1ᵇ	2.0ᵃ	0.6ᵃ	<0.001
Congestive heart failure^‡^	0.4	0.3	0.5	0.8	0.0	—
Peripheral arterial disease	0.6	0.7	0.8	0.8	0.2	0.625
Medication use (%)						
Diuretic	2.4	3.6ᵃ	1.1ᵃ	2.3ᵃ	0.4ᵇ	<0.001
ACE inhibitor or ARB	5.2	6.1ᵃ	6.9ᵃ	2.4ᵇ	4.0ᵃ	<0.001
α-blocker	0.8	0.3ᵃ	0.2ᵃ	2.5ᵇ	1.4ᶜ	<0.001
β-blocker	1.6	2.3	1.1	1.0	1.2	0.142
Calcium antagonist	9.9	14.0ᵃ	8.8ᵇ	5.8ᶜ	4.2ᶜ	<0.001
Other antihypertensive agent	7.6	8.2	7.8	8.6	4.7	0.093
Lipid lowering agent	3.2	3.6	3.5	3.2	1.6	0.197
Anti-platelet agent	4.6	7.5ᵃ	3.4ᵇ	1.6ᶜ	1.4ᶜ	<0.001
Oral anticoagulants	0.7	0.4	1.0	1.0	0.8	0.499
EQ- VAS score, mean (SE)	82.6 (0.2)	80.4 (0.3)ᵃ	82.9 (0.6)ᵇ	83.9 (0.5)ᵇ	86.4 (0.5)ᶜ	<0.001
EQ-5D utility score, mean (SE)	0.994 (0.001)	0.961 (0.003)ᵃ	0.963 (0.004)ᵃ	0.969 (0.004)ᵃ	0.994 (0.001)ᵇ	<0.001

ACE, angiotensin-converting enzyme; ARB, angiotensin receptor blocker; BMI, body mass index; DBP, diastolic blood pressure; SBP, systolic blood pressure. *p*-values are for overall comparisons across provinces (survey-weighted linear regression for continuous variables and Rao–Scott chi-square tests for categorical variables). For continuous variables, pairwise comparisons were conducted using Tukey–Kramer adjustment; for categorical variables, Bonferroni adjusted pairwise comparisons were applied. Different superscript letters (a, b, c, d) within a row indicate significant pairwise differences at *p* < 0.05. When the overall comparison was significant but no pairwise comparison remained significant after adjustment, only the overall *p*-value is shown without superscripts. ^*^ Ever smoking defined as smoking on most days for ≥1 year. ^†^ Currently smoking defined as smoking on most days in the past year. ^^^ SBP and DBP data were missing for five participants in Shanxi province. ^‡^ Not calculated due to small numbers.

Participants in Shanxi were significantly older (49.5 years) than those in the other provinces (44.5–47.5 years). BMI was highest in Heilongjiang (25.6 kg/m^2^) and lowest in Guangdong (23.8 kg/m^2^). The proportion of participants with junior high school education or above was lowest in Heilongjiang (14.9%) and highest in Guangdong (40.9%). Regarding blood pressure, Heilongjiang had higher mean SBP and DBP (136.4 mmHg; 84.9 mmHg) than Guizhou (126.3 mmHg; 80.6 mmHg) and Guangdong (127.4 mmHg; 80.8 mmHg). EQ-VAS scores were lowest in Shanxi (80.4) and highest in Guangdong (86.4). EQ-5D utility scores were modestly but significantly higher in Guangdong (0.994) than in the other provinces (0.961–0.969) (Table 1).

### 3.3 Dietary intake of sodium and potassium

The weighted mean sodium and potassium concentrations from spot urine samples were 155.1 mmol/L (SE 1.6) and 41.2 mmol/L (SE 0.5), respectively (Table 2). Based on 24-h urine collections, the weighted mean urinary sodium and potassium excretion was 5.4 g/day (SE 0.1) and 1.84 g/day (SE 0.03), respectively. Under the assumption of non-renal losses (7% for sodium and 23% for potassium), the estimated dietary intake of sodium and potassium from 24-h urine collections was 5.8 g/day (SE 0.1) and 2.39 g/day (SE 0.04), respectively. The weighted mean salt intake was 14.7 g/day (SE 0.2). Significant differences across provinces were observed (overall comparison, *p* < 0.001). Participants with and without eligible 24-h urine samples were generally comparable across most demographic and health-related characteristics. However, participants with eligible 24-h urine samples were less likely to be female, had lower smoking prevalence and waist circumference, and differed in provincial distribution compared with those without eligible urine samples (Supplementary Table S5).

**Table 2:** Weighted spot urine and 24-h urine among rural adults in four provinces of China.

Characteristics	Total	Shanxi	Heilongjiang	Guizhou	Guangdong	*p*-value
Spot urine						
Number (n)	2,629	1,192	479	479	479	
Sodium (mmol/L)	155.1 (1.6)	171.4 (2.2)^a^	153.2 (3.2)^b^	168.3 (4.3)^a^	102.9 (2.8)^c^	<0.001
Potassium (mmol/L)	41.2 (0.5)	41.5 (0.7)^b^	47.6 (1.3)^a^	43.3 (1.0)^b^	32.0 (1.0)^c^	<0.001
24-h urine						
Number (n)	1,601	599	274	388	340	
Urine sodium excretion (g/day)	5.4 (0.1)	6.4 (0.2)^a^	5.1 (0.2)^b^	5.6 (0.2)^b^	3.5 (0.1)^c^	<0.001
Sodium intake (g/day) ^*^	5.8 (0.1)	6.9 (0.2)^a^	5.5 (0.2)^b^	6.0 (0.2)^b^	3.8 (0.2)^c^	<0.001
Salt equivalent based on urine sodium (g/day)	13.6 (0.2)	16.3 (0.4)^a^	13.0 (0.5)^b^	14.2 (0.5)^b^	9.0 (0.4)^c^	<0.001
Salt intake (g/day) ^*^	14.7 (0.2)	17.5 (0.4)^a^	14.0 (0.6)^b^	15.2 (0.5)^b^	9.6 (0.4)^c^	<0.001
Urine potassium excretion (g/day)	1.84 (0.03)	1.95 (0.05)^a^	1.75 (0.07)^ab^	1.92 (0.07)^a^	1.67 (0.06)^b^	0.003
Potassium intake (g/day) ^*^	2.39 (0.04)	2.53 (0.06)^a^	2.27 (0.09)^ab^	2.49 (0.09)^a^	2.17 (0.08)^b^	0.003

*p-*values are from survey-weighted linear regression across provinces. Pairwise comparisons were conducted using Tukey–Kramer adjustment. Different superscript letters (a, b, c, d) within a row indicate significant pairwise differences at *p* < 0.05. ^*^ Estimated dietary sodium, potassium, and salt intakes were calculated from 24-h urine samples using correction factors for non-urinary losses (urinary sodium/0.93; urinary potassium/0.77).

There was substantial regional variation. In spot urine, Guangdong had markedly lower sodium (102.9 mmol/L) and potassium (32.0 mmol/L) concentrations than Shanxi, Heilongjiang, and Guizhou, while Heilongjiang exhibited the highest potassium concentration (47.6 mmol/L). Based on 24-h urine collections, Shanxi showed the highest sodium intake (6.9 g/day) and Guizhou showed the highest potassium intake (1.92 g/day) among the four provinces. Guangdong had the lowest sodium intake (3.8 g/day) and salt intake (9.6 g/day) among the four provinces (Table 2).

### 3.4 Salt-related knowledge, attitudes, and behaviours

Weighted analyses showed that for salt-related knowledge, most participants recognized that excessive salt intake is harmful (77.7%) and affects blood pressure (66.4%) and CVD (67.1%) (Table 3). Only 14.5% were aware of the recommended daily salt intake, but 31.3% had heard of potassium-enriched salt. The salt-related knowledge of participants varied significantly across provinces (overall comparison, *p* < 0.001). Participants in Heilongjiang showed the highest awareness of salt-related health risks (84.8%) but the lowest awareness of potassium-enriched salt (16.6%), and participants in Shanxi had the lowest proportion that knew of the recommended maximum daily salt intake (3.7%).

**Table 3:** Weighted salt-related knowledge, attitudes, and behaviours of participants (%).

Questions	Total	Shanxi	Heilongjiang	Guizhou	Guangdong	*p*-value
Number (n)	2,669	1,229	480	481	479	
Knowledge						
Aware that too much salt or salty sauce affects health	77.7	75.2ᶜ	84.8ᵃ	76.0ᵇ	78.7ᵇ	<0.001
Aware that reducing salt intake can lower blood pressure	66.4	63.1ᵇ	68.1ᵃ	67.5ᵃ	72.0ᵃ	<0.001
Aware that reducing salt intake can lower the risk of cardiovascular disease	67.1	60.4ᶜ	79.5ᵃ	65.3ᵇ	73.9ᵃ	<0.001
Aware of recommended daily salt intake	14.5	3.7ᶜ	12.8ᵇ	30.1ᵃ	28.3ᵃ	<0.001
Aware of potassium-enriched salt	31.3	34.5ᵃ	16.6ᶜ	29.6ᵇ	39.0ᵃ	<0.001
Attitude						
There is a need to reduce salt in purchased food	44.9	37.7ᶜ	55.5ᵃ	43.9ᵇ	53.6ᵃ	<0.001
Reducing salt in cooking is important	67.3	66.3ᵇ	83.6ᵃ	63.8ᵇ	56.9ᶜ	<0.001
Participant believes they consume too much salt	21.1	23.7ᵃ	19.4ᵃ	12.3ᵇ	25.0ᵃ	<0.001
Participant believes they consume too much salty sauce	18.5	20.6ᵃ	17.6ᵃ	8.4ᶜ	24.3ᵇ	<0.001
Participant believes that potassium-enriched salt is good for health^*^	19.0	19.2ᵇ	12.4ᶜ	20.2ᵇ	24.2ᵃ	<0.001
Participant believes that potassium-enriched salt is a healthy way to reduce sodium^*^	18.5	18.6ᵇ	11.2ᶜ	19.6ᵇ	24.2ᵃ	<0.001
Participant believes that potassium-enriched salt has a bad taste^*^	6.8	6.8ᵃ	4.3ᵇ	5.0ᶜ	11.5ᵃ	<0.001
Behaviour						
Currently use potassium-enriched salt^*^	12.8	12.6ᵇ	7.0ᶜ	12.3ᵇ	19.7ᵃ	<0.001
Consume food outside of the home	28.0	30.3ᵃ	19.9ᵇ	23.3ᵇ	35.2ᵃ	<0.001
Try to reduce salt intake	50.1	50.0ᵇ	64.7ᵃ	64.5ᵃ	50.9ᵇ	<0.001
Salt reduction was advised by doctor^†^	53.4	42.9ᵇ	62.4ᵃ	62.6ᵃ	62.1ᵃ	<0.001
Heard or participated in salt reduction health education^†^	59.4	60.0ᵇ	52.3ᶜ	52.7ᶜ	72.0ᵃ	<0.001

*p*-values are from Rao–Scott χ^2^ tests comparing provinces. Pairwise comparisons were adjusted using the Bonferroni method. Different superscript letters (a, b, c, d) within a row indicate statistically significant differences between provinces (*p* < 0.05). ^*^ Variables were asked only among participants who were aware of potassium-enriched salt. Proportions are presented using the total sample as the denominator to reflect population-level prevalence. ^†^ Variables reflecting external interventions (e.g., physician advice, health education) were categorized under Behaviour, although they are not core dietary behaviours, in order to capture participants’ engagement in salt-reduction activities.

For salt-related attitudes, 44.9% of participants agreed that reducing salt in purchased food is necessary, and 67.3% emphasized its importance in cooking, yet fewer than 25% believed their own intake was excessive. Only 19.0% of participants believed that potassium-enriched salt is good for health. Significant provincial differences in salt-related attitudes were observed (overall comparison, *p* < 0.001). Participants in Heilongjiang expressed stronger support for salt reduction in cooking (83.6%) compared with those in Shanxi (66.3%) and Guizhou (63.8%), while participants in Guizhou reported the lowest self-perceived excess intake (8.4%). Guangdong had the highest proportion of participants who perceived potassium-enriched salt as having a bad taste (11.5%).

For salt-related behaviours, half of participants (50.1%) reported trying to reduce salt intake. The most commonly reported practices were avoiding consumption of processed foods (94.5%) and using less salt in pickling (91.3%). Being more concerned about other nutrients than sodium intake was the most frequently cited barrier (44.9%) (Supplementary Table S6). Salt-related behaviours of participants also differed significantly across provinces (overall comparison, *p* < 0.001). Participants in Heilongjiang reported the lowest current use of potassium-enriched salt (7.0%), while participants in Guangdong had the highest exposure to salt-reduction education (72.0%). Detailed distributions of patterns of eating out-side the home and channels of salt-reduction health education are provided in Supplementary Table S7.

### 3.5 Dietary sources of sodium and potassium

**Figure 3:**
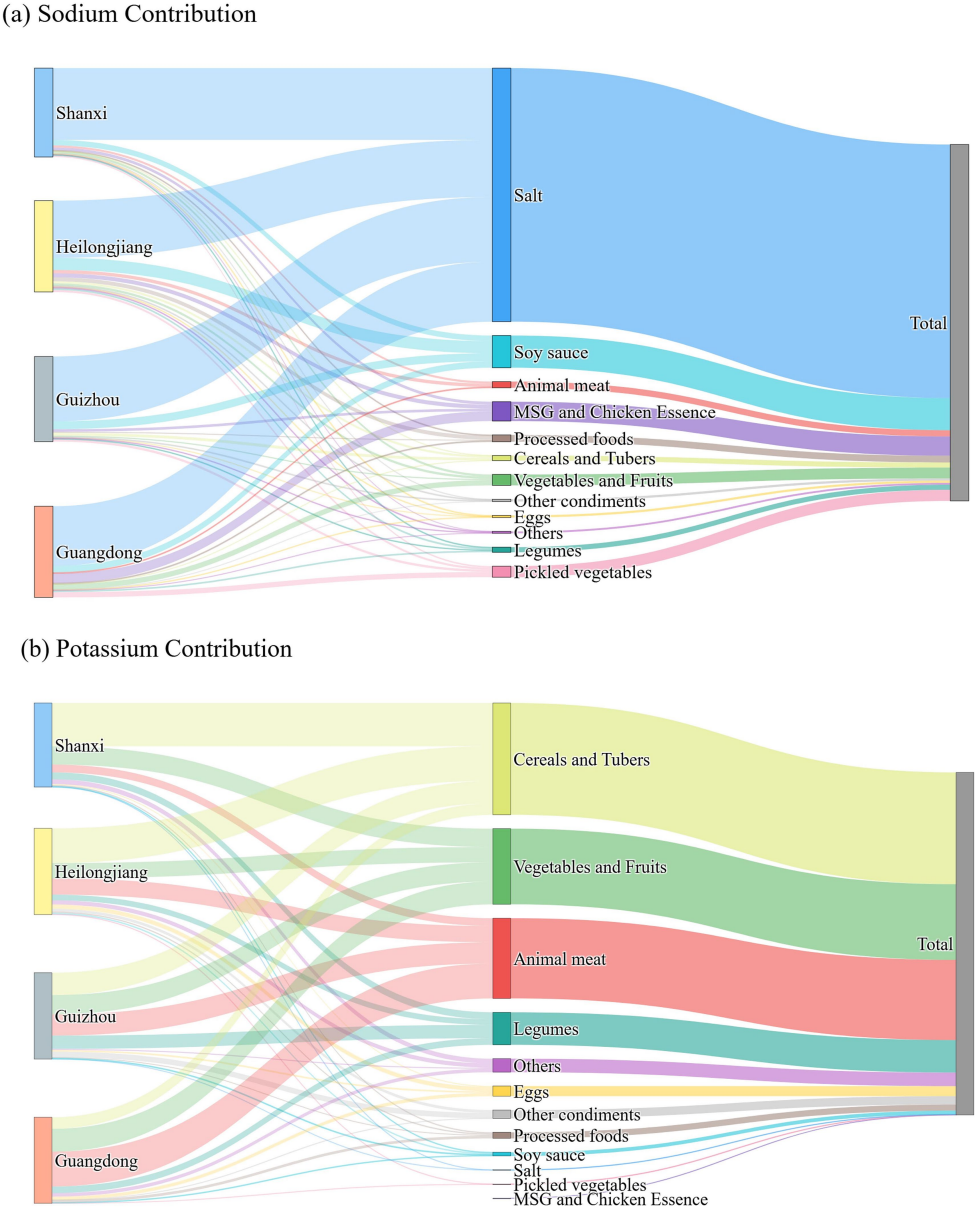
Weighted contribution of foods and beverages to daily sodium and potassium intake based on 24-h dietary recall: **(a)** Contribution of foods and beverages to daily sodium intake; **(b)** Contribution of foods and beverages to daily potassium intake.

Based on weighted estimates, Figure 3 illustrates the major sources of dietary sodium and potassium. Salt added during cooking was the predominant source of sodium, contributing 72.1% overall, followed by soy sauce (7.7%), animal meat (4.3%), and MSG and chicken essence (3.5%). Across provinces, salt added during cooking remained the leading contributor, accounting for 78.5% in Shanxi, 62.5% in Heilongjiang, 70.8% in Guizhou, and 65.5% in Guangdong.

For dietary potassium, cereals and tubers were the main source (37.3%), followed by vegetables and fruits (21.9%), animal meat (19.5%), and legumes (9.0%). Provincial differences were evident: participants in Shanxi, Heilongjiang, and Guizhou derived most potassium from cereals and tubers (50.2, 40.2, and 26.0%, respectively), whereas participants in Guangdong obtained the largest share from animal meat (40.5%), with cereals and tubers contributing only 13.4%. Detailed data are provided in Supplementary Table S8.

Dietary source analyses were based on participants with available 24-h dietary recall data. Participants with and without dietary recall data were generally comparable across most demographic and health-related characteristics, although participants with dietary recall data were slightly younger and had a higher proportion of individuals with junior high school education or above. Detailed data are provided in Supplementary Table S9.

## 4 Discussion

This cross-sectional study shows that dietary sodium intake among rural Chinese adults substantially exceeds recommendations, whereas dietary potassium intake falls well below recommended levels (2, 18). The high consumption levels of sodium show that while there was high awareness of the health risks of excessive salt intake and positive attitudes toward salt reduction, these did not translate into effective sodium reduction behaviours. We observed notable regional heterogeneity in dietary intake of sodium and potassium and associated KAB which suggest that there is opportunity for enhanced implementation of strategies targeting sodium and potassium intake, though additional work will be required to identify the mechanisms by which to achieve change.

The mean sodium intake among rural adults in our study, based on 24-h urine collections, was 5.8 g/day. This level is higher than the most recent national estimate for rural areas (4.4 g/day) using 24-h urine collection (11), indicating that sodium intake in the surveyed rural populations remains substantial. Importantly, the current intake remains approximately twice the WHO recommended maximum of <2.0 g/day (2). The predominant source of dietary sodium in our survey was salt added during home cooking. This is likely because salt in China is cheap, widely available, and home cooking remains the dominant dietary practice, particularly in rural areas (40). In many low- and middle-income countries (LMICs), household cooking salt contributes more than half of sodium intake, in sharp contrast to high-income countries where processed foods and restaurant meals are the dominant source of dietary sodium (41). In upper-middle-income countries such as China, sodium-reduction strategies should similarly prioritize household-level interventions rather than adopting approaches focused on processed and restaurant foods.

Mean potassium intake has remained low at 2.39 g/day, averaging only about 40% of the WHO recommended minimum (3.5 g/day) (18). Multiple studies in South Asia and sub-Saharan Africa, have also reported widespread potassium insufficiency (42). While cereals and tubers were the dominant source of potassium for participants in most provinces, they do not contain high concentrations of potassium and there was limited intake of potassium-rich foods such as fruits, vegetables, and legumes (37). Evidence suggests that economic constraints and affordability are important determinants of fresh produce consumption, especially in rural settings where lower household income and limited access to affordable healthy foods have been linked to reduced intake of fruits and vegetables (43). Moreover, seasonal variation in the availability of local produce has been shown to affect consumption patterns, with higher intake occurring during peak harvest seasons and lower intake during off-season periods (44). Notably, although EQ-5D utility and EQ-VAS scores were generally high in this rural population, excessive sodium intake and insufficient potassium intake remained highly prevalent. This may indicate that unhealthy dietary patterns can persist even among individuals reporting relatively good perceived health status. The relatively high HRQoL scores observed in this study may partly reflect the generally preserved daily functioning and self-care ability of this predominantly middle-aged rural population.

Regarding salt-related KAB, the high rates of knowledge about the health risks of excessive salt or salty sauce intake were consistent. The high level of awareness is likely attributable to prior public health education and salt-reduction campaigns (45). In contrast, the effective implementation of salt-reduction practices remained low. This finding has also been widely documented in other settings (46). Behaviour change frameworks such as the Health Belief Model and Social Cognitive Theory emphasize that the combination of self-efficacy, motivation, and supportive environments are critical for action. Awareness alone is typically insufficient and environments in rural China and other settings are generally not adequately supportive of behaviour change (47). The main strategy for reducing salt in national programs was the recommendation to limit the amount of salt added during cooking (48). Although this approach is simple and practical (49), its effectiveness is often limited because people are reluctant to sacrifice a salty taste (4, 17). Potassium-enriched salt may offer a feasible approach to this problem since it can be used as a one-to-one switch for regular salt with the retention of the same salty flavor for most people. Potassium-enriched salt also has the advantage of simultaneously lowering sodium and increasing potassium intake to maximize blood pressure reduction (50). The Salt Substitute and Stroke Study (SSaSS), a large cluster randomized controlled trial, confirmed cardiovascular benefits and high acceptability, with over 92% of participants still using the substitute after 5 years, underscoring its potential as a sustainable, population-level intervention (51).

Our study highlights substantial provincial disparities in dietary intake of sodium and potassium and salt-related KAB. In terms of dietary intake of sodium and potassium, participants in Shanxi showed the highest sodium intake (6.9 g/day), likely reflecting higher salt use and stronger salt taste preference. This aligns with previous large-scale evidence suggesting that sodium intake in central China may exceed that observed in northern and southern regions (52). Notably, sodium intake in Guizhou was also relatively high, which may be related to ethnic dietary customs emphasizing pickled vegetables, salt-heavy seasoning practices and a humid climate that increases the need for salt to prevent food spoilage, although these factors were not directly assessed in the present study (53). The highest potassium intake in Shanxi and Guizhou provinces (2.53 and 2.49 g/day respectively) may reflect the higher consumption of plant-based foods. In Shanxi, wheat-based staple foods, other cereals, and soybeans constitute a major component of the traditional diet (54), while in Guizhou, vegetable-rich dietary customs may further contribute to potassium intake (55). In contrast, participants in Guangdong had the lowest sodium intake (3.8 g/day), likely due to their preference for fresh and lightly seasoned foods (56). In terms of salt-related KAB, participants in Shanxi showed the lowest awareness of the recommended sodium limit (3.7%) and willingness to reduce salt (50.0%). Although our study did not directly evaluate provincial educational strategies, these findings may indicate insufficient dissemination of salt-reduction health education and a relatively low level of behavioural adoption. By comparison, participants in Heilongjiang had the highest reported salt-reduction behaviour (64.7%), which may be associated with higher levels of salt-related knowledge and risk perception observed in this population (4). However, salt intake levels in our study remained high, and the adoption of potassium-enriched salt was limited. These findings suggest that behavioural change may require additional approaches (51). Future research should explore the feasibility, accessibility, and potential implementation challenges of potassium-enriched salt, including possible contraindications for individuals with kidney disease or those taking potassium-retaining medications. Educational efforts should continue to focus on improving awareness of recommended limits and health risks, enhancing practical skills for salt reduction, and addressing environmental barriers such as product labeling, availability of low-salt foods, and access to potassium-enriched salts.

There are both strengths and limitations of this study. The strengths include the use of stratified random sampling by age and sex across four geographically diverse Chinese provinces, together with the application of population-based sampling weights to enhance demographic balance and the representativeness of the estimates. Moreover, we used 24-h urine samples, the gold standard for estimating sodium and potassium intake, and applied established corrections for non-urinary losses to improve accuracy. Nevertheless, several limitations exist. Firstly, the absence of qualitative data limits exploration of cultural and social drivers and the absence of data for urban populations limits comparisons between rural and urban settings. Secondly, the 24-h dietary recall data are self-reported and therefore subject to recall error, under-reporting of discretionary salt use, and potential social desirability bias, which may lead to misestimation of food source contributions. Thirdly, although four provinces representing different geographic regions of China were included, the study sites were purposively selected rather than randomly sampled. Therefore, the study was not designed to generate nationally representative estimates, and the selected study sites may differ from other rural areas in China in terms of socioeconomic characteristics, dietary habits, healthcare access, and salt-related practices. Accordingly, the findings and regional comparisons should be interpreted as descriptive of the sampled rural populations rather than population-level differences. Furthermore, because participant recruitment and field coordination involved local village healthcare personnel, some responses related to salt-related behaviours and health practices may have been subject to social desirability bias. Lastly, day-to-day variation in individual sodium intake was not captured, as only a single 24-h urine sample was collected per participant.

## 5 Conclusion

This study found that sodium intake remains excessive while potassium intake is insufficient among rural adult participants in four Chinese provinces. We also observed substantial regional variation in dietary intake and salt-related KAB. The findings suggest that ongoing education on sodium reduction and practical training in salt-lowering strategies may be valuable for informing future interventions, although causal effects cannot be inferred from this cross-sectional study. Practical tools and improved access to lower-sodium foods or healthier salt alternatives could be explored in future research or public health practice to enhance potential impact. In addition, potassium-enriched salt may represent a feasible approach to simultaneously reduce sodium intake and increase potassium intake in rural settings, but further studies are needed to evaluate its accessibility, acceptability, and long-term implementation. Importantly, as the study sites were purposively selected, the findings may not be nationally representative, and caution should be taken when generalizing the results beyond the sampled populations.

## Data Availability

The raw data supporting the conclusions of this article will be made available by the authors, without undue reservation.
