# Supplementary material for: Dietary intake and sources of sodium and potassium, and salt-related knowledge, attitudes, and behaviours among rural adults in China: a cross-sectional study
Source: Front Nutr. 2026 Jun 11;13:1861183. doi: 10.3389/fnut.2026.1861183 (PMC13293782; doi:10.3389/fnut.2026.1861183)
Supplement: Supplementary file 1 [file Table_1.DOCX]

Supplementary Material

# Supplementary Tables

Table S1. Comparison of questionnaire participants with the 2020 China Population Census (%).

Table S2. Comparison of spot urine participants with the 2020 China Population Census (%)

Table S3. Comparison of 24-hour urine participants with the 2020 China Population Census (%)

Table S4. Comparison of 24-hour dietary recall participants with the 2020 China Population Census (%)

Table S5. Comparison of characteristics between participants with eligible and ineligible 24-hour urine samples

Table S6. Weighted salt reduction practices and barriers in four provinces of China (%)

Table S7. Weighted patterns of eating outside the home and channels of salt reduction health education (%)

Table S8. Sources of dietary sodium and potassium intake by province (%)

Table S9. Comparison of characteristics between participants with and without 24-hour dietary recall

**Table S1. Comparison of questionnaire participants with the 2020 China Population Census (%)**

| **Variables** | | | **EVEREST** | | **The 2020 China Population Census** | | **Weight** |
| --- | --- | --- | --- | --- | --- | --- | --- |
| **Province** | **Sex** | **Age** | **N** | **%** | **N** | **%** |  |
| Shanxi | Male | 18-44 | 206 | 16.8 | 2,228,365 | 23.3 | 1.386 |
|  |  | 45-69 | 374 | 30.5 | 2,865,663 | 29.9 | 0.982 |
|  |  |  |  |  |  |  |  |
|  | Female | 18-44 | 234 | 19.1 | 1,814,344 | 18.9 | 0.993 |
|  |  | 45-69 | 415 | 33.8 | 2,684,108 | 28.0 | 0.829 |
|  |  |  |  |  |  |  |  |
| Heilongjiang | Male | 18-44 | 127 | 26.5 | 1,734,442 | 20.7 | 0.782 |
|  |  | 45-69 | 118 | 24.6 | 2,714,061 | 32.4 | 1.316 |
|  |  |  |  |  |  |  |  |
|  | Female | 18-44 | 124 | 25.9 | 1,307,791 | 15.6 | 0.604 |
|  |  | 45-69 | 111 | 23.2 | 2,629,901 | 31.2 | 1.356 |
|  |  |  |  |  |  |  |  |
| Guizhou | Male | 18-44 | 126 | 26.2 | 2,869,018 | 25.6 | 0.977 |
|  |  | 45-69 | 118 | 24.5 | 2,987,887 | 26.7 | 1.087 |
|  |  |  |  |  |  |  |  |
|  | Female | 18-44 | 113 | 23.5 | 2,487,786 | 22.2 | 0.945 |
|  |  | 45-69 | 124 | 25.8 | 2,861,485 | 25.6 | 0.991 |
|  |  |  |  |  |  |  |  |
| Guangdong | Male | 18-44 | 121 | 25.3 | 6,239,848 | 29.1 | 1.155 |
|  |  | 45-69 | 119 | 24.9 | 5,142,187 | 23.9 | 0.967 |
|  |  |  |  |  |  |  |  |
|  | Female | 18-44 | 124 | 25.9 | 5,122,532 | 23.9 | 0.925 |
|  |  | 45-69 | 115 | 24.1 | 4,889,248 | 22.8 | 0.952 |

**Table S2. Comparison of spot urine participants with the 2020 China Population Census (%)**

| **Variables** | | | **EVEREST** | | **The 2020 China Population Census** | | **Weight** |
| --- | --- | --- | --- | --- | --- | --- | --- |
| **Province** | **Sex** | **Age** | **N** | **%** | **N** | **%** |  |
| Shanxi | Male | 18-44 | 203 | 17.0 | 2,228,365 | 23.3 | 1.365 |
|  |  | 45-69 | 369 | 31.0 | 2,865,663 | 29.9 | 0.967 |
|  |  |  |  |  |  |  |  |
|  | Female | 18-44 | 213 | 17.9 | 1,814,344 | 18.9 | 1.059 |
|  |  | 45-69 | 407 | 34.1 | 2,684,108 | 28.0 | 0.820 |
|  |  |  |  |  |  |  |  |
| Heilongjiang | Male | 18-44 | 127 | 26.5 | 1,734,442 | 20.7 | 0.781 |
|  |  | 45-69 | 118 | 24.6 | 2,714,061 | 32.4 | 1.316 |
|  |  |  |  |  |  |  |  |
|  | Female | 18-44 | 124 | 25.9 | 1,307,791 | 15.6 | 0.603 |
|  |  | 45-69 | 110 | 23.0 | 2,629,901 | 31.2 | 1.360 |
|  |  |  |  |  |  |  |  |
| Guizhou | Male | 18-44 | 126 | 26.3 | 2,869,018 | 25.6 | 0.973 |
|  |  | 45-69 | 117 | 24.4 | 2,987,887 | 26.7 | 1.091 |
|  |  |  |  |  |  |  |  |
|  | Female | 18-44 | 113 | 23.6 | 2,487,786 | 22.2 | 0.940 |
|  |  | 45-69 | 123 | 25.7 | 2,861,485 | 25.6 | 0.995 |
|  |  |  |  |  |  |  |  |
| Guangdong | Male | 18-44 | 121 | 25.3 | 6,239,848 | 29.1 | 1.150 |
|  |  | 45-69 | 119 | 24.8 | 5,142,187 | 23.9 | 0.964 |
|  |  |  |  |  |  |  |  |
|  | Female | 18-44 | 124 | 25.9 | 5,122,532 | 23.9 | 0.921 |
|  |  | 45-69 | 115 | 24.0 | 4,889,248 | 22.8 | 0.948 |

**Table S3. Comparison of 24-hour urine participants with the 2020 China Population Census (%)**

| **Variables** | | | **EVEREST** | | **The 2020 China Population Census** | | **Weight** |
| --- | --- | --- | --- | --- | --- | --- | --- |
| **Province** | **Sex** | **Age** | **N** | **%** | **N** | **%** |  |
| Shanxi | Male | 18-44 | 79 | 13.2 | 2,228,365 | 23.3 | 1.763 |
|  |  | 45-69 | 174 | 29.1 | 2,865,663 | 29.9 | 1.030 |
|  |  |  |  |  |  |  |  |
|  | Female | 18-44 | 106 | 17.7 | 1,814,344 | 18.9 | 1.069 |
|  |  | 45-69 | 240 | 40.1 | 2,684,108 | 28.0 | 0.699 |
|  |  |  |  |  |  |  |  |
| Heilongjiang | Male | 18-44 | 65 | 23.7 | 1,734,442 | 20.7 | 0.873 |
|  |  | 45-69 | 58 | 21.2 | 2,714,061 | 32.4 | 1.532 |
|  |  |  |  |  |  |  |  |
|  | Female | 18-44 | 75 | 27.4 | 1,307,791 | 15.6 | 0.571 |
|  |  | 45-69 | 76 | 27.7 | 2,629,901 | 31.2 | 1.126 |
|  |  |  |  |  |  |  |  |
| Guizhou | Male | 18-44 | 92 | 23.7 | 2,869,018 | 25.6 | 1.079 |
|  |  | 45-69 | 91 | 23.5 | 2,987,887 | 26.7 | 1.137 |
|  |  |  |  |  |  |  |  |
|  | Female | 18-44 | 98 | 25.3 | 2,487,786 | 22.2 | 0.878 |
|  |  | 45-69 | 107 | 27.6 | 2,861,485 | 25.6 | 0.927 |
|  |  |  |  |  |  |  |  |
| Guangdong | Male | 18-44 | 78 | 22.9 | 6,239,848 | 29.1 | 1.267 |
|  |  | 45-69 | 79 | 23.2 | 5,142,187 | 23.9 | 1.030 |
|  |  |  |  |  |  |  |  |
|  | Female | 18-44 | 97 | 28.5 | 5,122,532 | 23.9 | 0.836 |
|  |  | 45-69 | 86 | 25.3 | 4,889,248 | 22.8 | 0.899 |

**Table S4. Comparison of 24-hour dietary recall participants with the 2020 China Population Census (%)**

| **Variables** | | | **EVEREST** | | **The 2020 China Population Census** | | **Weight** |
| --- | --- | --- | --- | --- | --- | --- | --- |
| **Province** | **Sex** | **Age** | **N** | **%** | **N** | **%** |  |
| Shanxi | Male | 18-44 | 72 | 21.8 | 2,228,365 | 23.3 | 1.066 |
|  |  | 45-69 | 91 | 27.6 | 2,865,663 | 29.9 | 1.085 |
|  |  |  |  |  |  |  |  |
|  | Female | 18-44 | 75 | 22.7 | 1,814,344 | 18.9 | 0.832 |
|  |  | 45-69 | 92 | 27.9 | 2,684,108 | 28.0 | 1.004 |
|  |  |  |  |  |  |  |  |
| Heilongjiang | Male | 18-44 | 32 | 25.0 | 1,734,442 | 20.7 | 0.829 |
|  |  | 45-69 | 32 | 25.0 | 2,714,061 | 32.4 | 1.297 |
|  |  |  |  |  |  |  |  |
|  | Female | 18-44 | 36 | 28.1 | 1,307,791 | 15.6 | 0.555 |
|  |  | 45-69 | 28 | 21.9 | 2,629,901 | 31.2 | 1.428 |
|  |  |  |  |  |  |  |  |
| Guizhou | Male | 18-44 | 33 | 26.8 | 2,869,018 | 25.6 | 0.954 |
|  |  | 45-69 | 28 | 22.8 | 2,987,887 | 26.7 | 1.171 |
|  |  |  |  |  |  |  |  |
|  | Female | 18-44 | 28 | 22.8 | 2,487,786 | 22.2 | 0.974 |
|  |  | 45-69 | 34 | 27.6 | 2,861,485 | 25.6 | 0.925 |
|  |  |  |  |  |  |  |  |
| Guangdong | Male | 18-44 | 32 | 25.2 | 6,239,848 | 29.1 | 1.153 |
|  |  | 45-69 | 32 | 25.2 | 5,142,187 | 23.9 | 0.950 |
|  |  |  |  |  |  |  |  |
|  | Female | 18-44 | 32 | 25.2 | 5,122,532 | 23.9 | 0.947 |
|  |  | 45-69 | 31 | 24.4 | 4,889,248 | 22.8 | 0.932 |

**Table S5. Comparison of characteristics between participants with eligible and ineligible 24-hour urine samples**

| **Characteristics** | **Participants with eligible 24-hour urine samples** | **Participants without eligible 24-hour urine samples**^*^ | **P-value** |
| --- | --- | --- | --- |
| Number (n) | 1601 | 1068 |  |
| Province (%) |  |  | <0.001 |
| Shanxi | 37.5 | 59.0 |  |
| Heilongjiang | 17.3 | 19.2 |  |
| Guizhou | 24.2 | 8.7 |  |
| Guangdong | 21.0 | 13.0 |  |
| Sex, Female (%) | 48.0 | 57.9 | <0.001 |
| Age (years), mean (SE) | 47.8 (0.3) | 47.5 (0.4) | 0.638 |
| 18 – 44 (%) | 39.4 | 41.2 | 0.356 |
| 45 – 69 (%) | 60.6 | 58.8 |  |
| BMI (kg/m^2^), mean (SE) | 24.7 (0.1) | 24.7 (0.1) | 0.784 |
| < 18.5 (%) | 2.7 | 3.7 | 0.419 |
| 18.5 – 24.0 (%) | 42.0 | 41.3 |  |
| 24.0 – 28.0 (%) | 39.7 | 38.3 |  |
| ≥ 28.0 (%) | 15.6 | 16.7 |  |
| Education (%) |  |  | 0.965 |
| Primary school and lower | 73.8 | 73.7 |  |
| Junior high school and above | 26.2 | 26.3 |  |
| Smoking (%) |  |  |  |
| Never smoked | 67.6 | 62.9 | 0.016 |
| Ever smoking^†^ | 32.2 | 36.8 | 0.016 |
| Current smoking^^^ | 29.2 | 35.2 | 0.002 |
| Waist circumference (cm), mean (SE) | 85.5 (0.3) | 86.9 (0.3) | 0.002 |
| SBP (mm Hg), mean (SE) ^‡^ | 129.1 (0.5) | 129.0 (0.6) | 0.868 |
| < 130 (%) | 56.7 | 58.0 | 0.518 |
| ≥130 (%) | 43.3 | 42.0 |  |
| DBP (mm Hg), mean (SE) ^‡^ | 81.7 (0.3) | 82.2 (0.4) | 0.275 |
| < 80 (%) | 45.1 | 44.5 | 0.745 |
| ≥ 80 (%) | 54.9 | 55.5 |  |
| Disease history (%) |  |  |  |
| Hypertension | 2.4 | 3.2 | 0.236 |
| Diabetes mellitus | 1.8 | 2.4 | 0.352 |
| Transient ischemic attack | 0.3 | 0.5 | 0.375 |
| Ischemic heart disease | 0.8 | 0.4 | 0.287 |
| Congestive heart failure^‡^ | 22.2 | 21.0 | 0.478 |
| Peripheral arterial disease | 6.1 | 5.5 | 0.533 |
| Medication use (%) |  |  |  |
| Diuretic | 2.3 | 2.5 | 0.681 |
| ACE inhibitor or ARB | 4.7 | 6.0 | 0.165 |
| α-blocker | 1.1 | 0.5 | 0.158 |
| β-blocker | 1.6 | 1.7 | 0.762 |
| Calcium antagonist | 9.5 | 10.4 | 0.485 |
| Other antihypertensive agent | 7.8 | 7.3 | 0.661 |
| Lipid lowering agent | 2.9 | 3.5 | 0.394 |
| Anti-platelet agent | 4.0 | 5.6 | 0.068 |
| Oral anticoagulants | 0.6 | 0.8 | 0.762 |
| EQ- VAS score, mean (SE) | 82.4 (0.3) | 82.8 (0.4) | 0.4346 |
| EQ-5D utility score, mean (SE) | 0.970 (0.001) | 0.967 (0.002) | 0.4246 |

^Note: Abbreviations: ACE, angiotensin-converting enzyme; ARB, angiotensin receptor blocker; BMI, body mass index; DBP, diastolic blood pressure; SBP, systolic blood pressure. P-values are for overall comparisons between groups and were derived from survey-weighted linear regression for continuous variables and Rao–Scott chi-square tests for categorical variables. * Participants without eligible 24‑h urine samples comprised those who did not provide a sample (due to menstruation, illness, or personal refusal) and those whose samples failed to meet eligibility criteria.^ ^† Ever smoking defined as smoking on most days for ≥1 year. ^ Currently smoking defined as smoking on most days in the past year. ‡ SBP and DBP data were missing for five participants in Shanxi province.^

**Table S6. Weighted salt reduction practices and barriers in four provinces of China (%)**

| **Questions** | **Total** | **Shanxi** | **Heilongjiang** | **Guizhou** | **Guangdong** | ***P*-value** |
| --- | --- | --- | --- | --- | --- | --- |
| **Practices for salt reduction** | | | | | | |
| Number (n) | 1329 | 621 | 304 | 168 | 236 |  |
| Check food labels | 89.6 | 54.4ᵃ | 90.8ᵇ | 84.8ᶜ | 89.6ᵈ | <0.001 |
| Buy low salt food | 62.8 | 13.5ᵃ | 49.1ᵃ | 44.1ᵇ | 71.9ᶜ | <0.001 |
| Use seasonings instead of salt | 51.6 | 15.5ᵃ | 57.0ᵇ | 49.1ᶜ | 72.3ᵃᵇ | <0.001 |
| Use salt-restricted spoon | 44.5 | 16.7ᵃ | 51.6ᵇ | 44.5ᶜ | 62.8ᵃᶜ | <0.001 |
| Use less salt in pickling | 91.3 | 49.3ᵃ | 90.2ᵇ | 74.9ᵃ | 91.3ᵇ | <0.001 |
| Avoid consumption of processed foods | 94.5 | 69.5ᵃ | 94.3ᵃ | 88.1ᵇ | 94.5ᵃ | <0.001 |
| Avoid eating out | 65.2 | 25.9ᵃ | 67.2ᵇ | 47.8ᵃ | 80.2ᵇ | <0.001 |
| Avoid putting salt shaker on the table | 65.2 | 23.9ᵃ | 80.5ᵇ | 74.2ᶜ | 65.2ᵇᶜ | <0.001 |
| Avoid adding salt to cooking | 65.2 | 49.3ᵃ | 67.2ᵇ | 74.2ᶜ | 80.2ᵈ | <0.001 |
| **Factors affecting adoption of salt reduction** | | | | | | |
| Number (n) | 1340 | 608 | 176 | 313 | 243 |  |
| Insufficient time | 22.4 | 12.2ᵃ | 40.3ᵇ | 29.0ᶜ | 27.5ᵈ | <0.001 |
| Do not want to reduce salt | 10.1 | 2.7ᵃ | 25.9ᵇ | 10.5ᶜ | 16.8ᵈ | <0.001 |
| Do not know how to judge excessive salt/sodium from food labels | 23.1 | 9.2ᵃ | 45.6ᵇ | 25.8ᶜ | 39.0ᵈ | <0.001 |
| Do not know the salt content of regular foods | 39.6 | 41.2ᵃ | 52.7ᵇ | 30.9ᶜ | 37.6ᵃ | <0.001 |
| Do not know how to make food taste good without salt | 28.6 | 11.5ᵃ | 54.3ᵇ | 31.0ᶜ | 50.9ᵇ | <0.001 |
| Low-sodium food is too expensive | 29.6 | 15.1ᵃ | 56.3ᵇ | 30.5ᶜ | 46.5ᵈ | <0.001 |
| Low-sodium food options are limited when purchasing food or dining out | 31.6 | 18.8ᵃ | 52.7ᵇ | 34.2ᶜ | 46.2ᵈ | <0.001 |
| Dislike the taste of low-sodium foods | 14.6 | 3.2ᵃ | 30.0ᵇ | 19.4ᶜ | 26.8ᵈ | <0.001 |
| The doctor does not recommend reducing sodium intake | 18.9 | 7.6ᵃ | 47.8ᵇ | 15.8ᶜ | 31.5ᵈ | <0.001 |
| More concerned about other nutrients than sodium intake | 44.9 | 51.5ᵃ | 54.4ᵃ | 32.5ᵇ | 37.1ᶜ | <0.001 |

Notes: *P*-values are from Rao–Scott χ² tests comparing provinces. Pairwise comparisons were adjusted using the Bonferroni method. Different superscript letters (a, b, c, d) within a row indicate statistically significant differences between provinces (P < 0.05). *Multiple answers were possible.

**Table S7. Weighted patterns of eating outside the home and channels of salt reduction health education (%)**

| **Questions** | **Total** | **Shanxi** | **Heilongjiang** | **Guizhou** | **Guangdong** | ***P*-value** |
| --- | --- | --- | --- | --- | --- | --- |
| **Patterns of eating outside the home (time** ≥ 2/week) | | | | | | |
| Number (n) | 756 | 375 | 103 | 115 | 163 |  |
| Fast food shop | 7.8 | 3.4ᵃ | 9.6ᵇ | 8.5ᶜ | 16.3ᵈ | <0.001 |
| Street food | 9.7 | 9.0ᵃ | 0.9ᵇ | 11.2ᶜ | 15.6ᵈ | 0.001 |
| Canteens | 23.8 | 24.5ᵃ | 12.6ᵇ | 38.8ᶜ | 18.6ᵃᵇ | <0.001 |
| Restaurants | 30.6 | 35.9ᵃ | 28.7ᵇ | 27.0ᶜ | 22.2ᵈ | 0.010 |
| Commissary/supermarket | 5.3 | 5.5 | 6.7 | 5.3 | 3.8 | 0.746 |
| Takeaways | 4.2 | 3.1 | 0.0 | 5.3 | 8.2 | 0.978 |
| **channels of salt reduction health education** | | | | | | |
| Number (n) | 1587 | 743 | 246 | 254 | 344 |  |
| Brochures or leaflets | 27.1 | 19.9ᵃ | 43.1ᵇ | 42.8ᵇ | 19.4ᵃ | <0.001 |
| Television/radio public service announcements | 54.6 | 47.7ᵃ | 53.4ᵇ | 51.3ᵇ | 72.7ᶜ | <0.001 |
| Social platforms | 39.0 | 34.9ᵃ | 39.0ᵃᵇ | 25.0ᶜ | 58.1ᵇ | <0.001 |
| Newspapers and magazines | 13.9 | 6.7ᵃ | 18.2ᵇ | 13.8ᵃ | 26.2ᶜ | <0.001 |
| Banners and blackboards | 12.8 | 7.4ᵃ | 25.7ᵇ | 17.9ᶜ | 10.9ᵃᶜ | <0.001 |
| Health workers | 50.9 | 48.0ᵃ | 54.6ᵇ | 71.3ᶜ | 39.2ᵃ | <0.001 |
| Family or friends | 27.9 | 25.7ᵃ | 30.6ᵇ | 19.3ᶜ | 36.9ᵈ | <0.001 |

Notes: *P*-values are from Rao–Scott χ² tests comparing provinces. Pairwise comparisons were adjusted using the Bonferroni method. Different superscript letters (a, b, c, d) within a row indicate statistically significant differences between provinces (P < 0.05). *Multiple answers were possible.

**Table S8. Sources of dietary sodium and potassium intake by province (%)**

| **Source** | **Total** | **Shanxi** | **Heilongjiang** | **Guizhou** | **Guangdong** |
| --- | --- | --- | --- | --- | --- |
| Number (n) | 708 | 330 | 128 | 123 | 127 |
| **Sodium** | | | | | |
| Salt | 72.1 | 78.5 | 62.5 | 70.8 | 65.5 |
| Soy sauce | 7.7 | 6.1 | 13.3 | 8.7 | 7.1 |
| Animal meat | 4.3 | 3.2 | 4.5 | 2.8 | 10.6 |
| MSG and Chicken Essence | 3.5 | 2.2 | 3.6 | 0.0 | 1.0 |
| Processed foods | 2.8 | 2.4 | 2.5 | 1.2 | 5.9 |
| Vegetables and Fruits | 2.7 | 1.6 | 2.2 | 2.6 | 5.7 |
| Cereals and Tubers | 0.0 | 0.9 | 4.3 | 1.2 | 1.0 |
| Others | 0.7 | 0.9 | 0.6 | 0.2 | 0.6 |
| Eggs | 1.3 | 0.9 | 2.1 | 0.8 | 1.8 |
| Other condiments | 1.4 | 0.1 | 2.3 | 3.0 | 0.1 |
| Legumes | 0.0 | 0.3 | 1.0 | 1.0 | 0.1 |
| Pickled vegetables | 0.4 | 0.1 | 1.0 | 0.7 | 0.3 |
| **Potassium** | | | | | |
| Cereals and Tubers | 37.3 | 50.2 | 40.2 | 26.0 | 13.4 |
| Vegetables and Fruits | 21.9 | 21.3 | 17.8 | 22.2 | 26.5 |
| Animal meat | 19.5 | 9.0 | 18.9 | 24.7 | 40.5 |
| Legumes | 9.0 | 8.0 | 6.6 | 15.5 | 7.7 |
| Others | 4.4 | 5.5 | 4.8 | 1.3 | 4.0 |
| Eggs | 3.0 | 1.1 | 4.9 | 1.9 | 3.8 |
| Other condiments | 2.2 | 0.0 | 3.3 | 6.3 | 0.1 |
| Processed foods | 1.7 | 1.5 | 1.5 | 1.1 | 3.0 |
| Soy sauce | 1.0 | 0.8 | 1.7 | 1.0 | 0.8 |
| Salt | 0.1 | 0.2 | 0.0 | 0.1 | 0.0 |
| Pickled vegetables | 0.0 | 0.0 | 0.3 | 0.0 | 0.1 |
| MSG and Chicken Essence | 0.0 | 0.0 | 0.0 | 0.0 | 0.0 |

Notes: Contribution of different food sources to sodium intake across provinces. Values are presented as descriptive percentages and were not subjected to statistical testing, as the primary purpose was to illustrate overall source distribution rather than assess between-province differences.

**Table S9. Comparison of characteristics between participants with and without 24-hour dietary recall**

| **Characteristics** | **Participants with 24-hour dietary recall^*^** | **Participants without 24-hour dietary recall** | **P-value** |
| --- | --- | --- | --- |
| Number (n) | 706 | 1963 |  |
| Province (%) |  |  | 0.965 |
| Shanxi | 46.7 | 45.9 |  |
| Heilongjiang | 18.1 | 18.1 |  |
| Guizhou | 17.4 | 18.2 |  |
| Guangdong | 17.9 | 17.8 |  |
| Sex, Female (%) | 53.0 | 51.6 | 0.536 |
| Age (years), mean (SE) | 46.1 (0.5) | 48.2 (0.3) | <0.001 |
| 18 – 44 (%) | 44.0 | 38.7 | 0.013 |
| 45 – 69 (%) | 56.0 | 61.3 |  |
| BMI (kg/m^2^), mean (SE) | 24.7 (0.2) | 24.7 (0.1) | 0.773 |
| < 18.5 (%) | 3.0 | 3.1 | 0.363 |
| 18.5 – 24.0 (%) | 44.5 | 40.8 |  |
| 24.0 – 28.0 (%) | 37.8 | 39.6 |  |
| ≥ 28.0 (%) | 14.7 | 16.5 |  |
| Education (%) |  |  | 0.031 |
| Primary school and lower | 70.7 | 74.8 |  |
| Junior high school and above | 29.3 | 25.2 |  |
| Smoking (%) |  |  |  |
| Never smoked | 66.4 | 65.5 | 0.653 |
| Ever smoking^†^ | 33.4 | 34.2 | 0.708 |
| Current smoking^^^ | 30.7 | 31.9 | 0.561 |
| Waist circumference (cm), mean (SE) | 85.7 (0.4) | 86.2 (0.3) | 0.319 |
| SBP (mm Hg), mean (SE) ^‡^ | 127.9 (0.8) | 129.4 (0.5) | 0.086 |
| < 130 (%) | 59.7 | 56.3 | 0.133 |
| ≥130 (%) | 40.3 | 43.7 |  |
| DBP (mm Hg), mean (SE) ^‡^ | 81.5 (0.5) | 82.1 (0.3) | 0.217 |
| < 80 (%) | 47.5 | 43.9 | 0.109 |
| ≥ 80 (%) | 52.5 | 56.1 |  |
| Disease history (%) |  |  |  |
| Hypertension | 1.8 | 3.0 | 0.105 |
| Diabetes mellitus | 1.8 | 2.1 | 0.623 |
| Transient ischemic attack | 0.5 | 0.4 | 0.766 |
| Ischemic heart disease | 0.4 | 0.7 | 0.430 |
| Congestive heart failure^‡^ | 20.2 | 22.3 | 0.249 |
| Peripheral arterial disease | 4.5 | 6.4 | 0.079 |
| Medication use (%) |  |  |  |
| Diuretic | 2.9 | 2.2 | 0.323 |
| ACE inhibitor or ARB | 5.5 | 5.1 | 0.717 |
| α-blocker | 1.0 | 0.8 | 0.681 |
| β-blocker | 0.8 | 1.9 | 0.055 |
| Calcium antagonist | 8.3 | 10.4 | 0.132 |
| Other antihypertensive agent | 6.3 | 8.0 | 0.164 |
| Lipid lowering agent | 3.0 | 3.2 | 0.740 |
| Anti-platelet agent | 4.9 | 4.5 | 0.423 |
| Oral anticoagulants | 0.7 | 0.6 | 0.853 |
| EQ- VAS score, mean (SE) | 83.0 (0.5) | 82.4 (0.3) | 0.299 |
| EQ-5D utility score, mean (SE) | 0.971 (0.003) | 0.968 (0.002) | 0.415 |

^Note: Abbreviations: ACE, angiotensin-converting enzyme; ARB, angiotensin receptor blocker; BMI, body mass index; DBP, diastolic blood pressure; SBP, systolic blood pressure. P-values are for overall comparisons between groups and were derived from survey-weighted linear regression for continuous variables and Rao–Scott chi-square tests for categorical variables. * A total of 708 participants completed the 24-hour dietary recall survey; however, two participants were excluded from this analysis due to missing baseline questionnaire information.^ ^† Ever smoking defined as smoking on most days for ≥1 year. ^ Currently smoking defined as smoking on most days in the past year. ‡ SBP and DBP data were missing for five participants in Shanxi province.^
